# A conundrum resolved: regulation and activation of UvrD-family DNA helicases/translocases

**DOI:** 10.1016/j.tibs.2026.02.005

**Published:** 2026-03-16

**Authors:** Timothy M. Lohman, Kacey N. Mersch, Ankita Chadda, Binh Nguyen, Eric A. Galburt

**Affiliations:** 1 Department of Biochemistry and Molecular Biophysics, Washington University in St Louis School of Medicine, St Louis, MO 63110, USA

## Abstract

Superfamily 1 helicases are conserved nonhexameric ATP-dependent enzymes that unwind DNA and RNA duplexes processively or remove proteins, playing critical roles in DNA repair, replication, recombination, and RNA processing. While crystal structures of Superfamily 1A UvrD-family helicases suggested that monomers are active helicases requiring an essential 2B regulatory domain–DNA interaction, biochemical studies show that helicase activation requires dimerization. Recent cryo-electron microscopy (EM) structures of *Mycobacterium tuberculosis* UvrD1 dimers reveal that dimerization involves the 2B domains, eliminating their inhibitory interaction with duplex DNA, contradicting these original models. *Escherichia coli* UvrD dimers use the same dimerization interface, suggesting a general mechanism for this class of helicases. Herein, we describe how these results require re-evaluation of helicase mechanisms that were based on the monomeric structures alone.

## Helicases are nucleic acid motors

**Figure 1.**
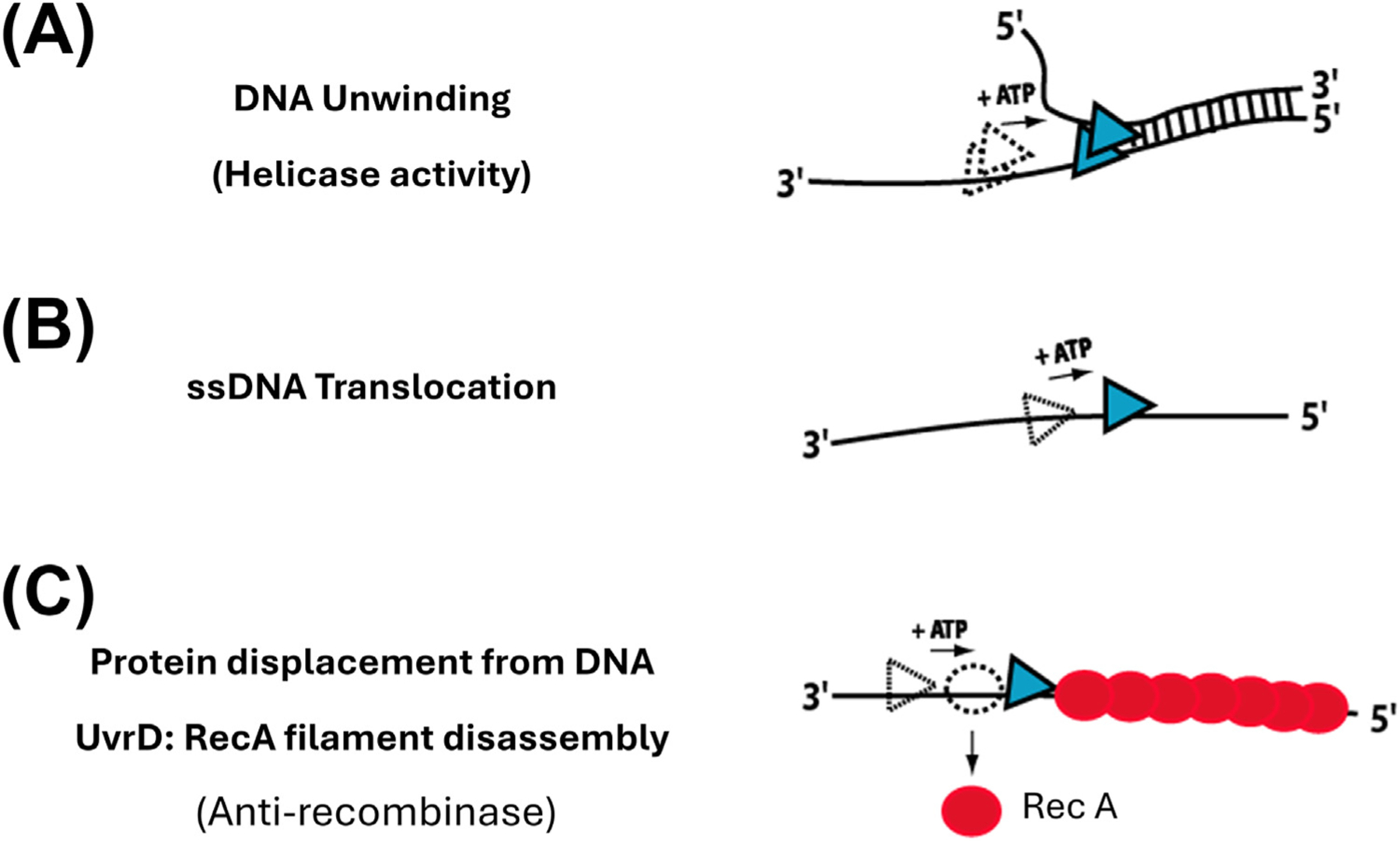
Three separable activities of SF1A motor proteins. (A) ATP-dependent DNA unwinding (helicase activity). (B) ATP-dependent unidirectional ssDNA translocation. (C) ATP-dependent displacement of proteins from ssDNA. SF1A: superfamily 1A; ssDNA: single-stranded DNA.

**Helicases** (see Glossary) are ubiquitous enzymes that play essential roles in all aspects of DNA and RNA metabolism [1–5]. Although there has been much progress in identifying the processes in which many of these enzymes function, there are still many questions surrounding the detailed mechanisms by which they function and are regulated. Whereas helicases were named for their ability to catalyze the unwinding of helical duplex DNA in ATP-dependent reactions [6], these enzymes often have multiple, separable activities, including DNA unwinding, unidirectional translocation along single-stranded (ss) DNA [7,8], displacement of DNA-bound proteins [9–11], and pushing proteins along DNA [12,13] (Figure 1).

An important but often neglected aspect of these enzymes is that they can self-assemble to form a variety of oligomeric states that regulate their functions [2,14–17]. In fact, the same oligomeric state is often not responsible for all functions of the enzyme. This is exemplified by the superfamily 1A (SF1A) DNA helicases, *Escherichia coli* Rep, *E. coli* UvrD, and *Bacillus stearothermophilus* PcrA. In the absence of **accessory proteins**, these enzymes must dimerize to unwind DNA, whereas monomers are capable of unidirectional (3′ to 5′) translocation along ssDNA [8,18–22]. Although the need for dimerization to activate these helicases has been known for over 25 years [16,17,20,23–25], the structural basis for this requirement and its consequences has remained unknown until recently. This is due to the fact that only structures of the monomeric forms of these enzymes had been obtained [26–30], leading to the controversial inference that the monomer is the active helicase [27,30].

Recent cryo-EM studies of the SF1A helicase UvrD1 from *Mycobacterium tuberculosis* [31] have finally revealed the structural basis for dimerization and activation of this class of helicases. These structures suggest that the previous monomeric–DNA junction structures [27,30] actually represent autoinhibited enzymes and that dimerization relieves this inhibition via a major rotation of a regulatory domain (2B domain) away from the duplex DNA in order to dimerize with the 2B domain of the other subunit. The 2B domain is regulatory, based on the observation that its deletion activates a helicase monomer [19]. Monomers of these helicases can also be activated by accessory proteins (MutL for UvrD [32,33], PriC for Rep [34], and RepD for PcrA [35]), and recent studies of UvrD show that this activation also involves an interaction with and rotation of the 2B regulatory domain [32,36]. Herein, we review the biochemical and structural studies of these SF1A helicases and re-evaluate the proposed mechanisms of DNA unwinding. Such mechanistic details are important to understand how these motor proteins function and may contribute to their use as potential therapeutic targets.

## Helicase superfamilies

Helicases are classified into six superfamilies (SF) [37]. SF3–6 comprise hexameric helicases [38,39], whereas SF1 helicases [40,41] and SF2 helicases are nonhexameric [15,42]. The main replicative helicases in most organisms are of the hexameric kind [39,43], whereas the SF1/2 helicases function in DNA repair, recombination [44], and RNA metabolism [3]. Although SF1 and SF2 helicases have often been assumed to function as monomers, these enzymes can self-assemble into higher oligomeric states; hence, the term nonhexameric [15] is appropriate. In fact, the assembly states populated by many of these enzymes have often not been characterized, especially when bound to DNA.

Our focus is on SF1 helicases, although some of what we consider, such as the role of oligomerization in function, pertains to SF2 helicases as well [45–47]. The SF1 helicases are **processive enzymes** and have been grouped into three subfamilies: UvrD-like, Pif1-like, and Upf1-like [42]. These are further divided based on their directionality of ATP-dependent translocation along ssDNA (i.e., SF1A helicases have 3′ to 5′ directionality, and SF1B helicases have 5′ to 3′ directionality) [38]. The Pif1-like and Upf1-like enzymes are SF1B helicases, whereas the UvrD-like enzymes, which we consider here, are SF1A helicases. As noted previously, the most well-studied members of the UvrD-like family not only include *E. coli* UvrD, *E. coli* Rep, and *B. stearothermophilus* PcrA but also include *Saccharomyces cerevisiae* Srs2 [48,49], *M. tuberculosis* UvrD1 [21,31,50,51], bacterial HelD [52], RecB within the RecBCD helicase [53], and human F-box helicase 1 (FBH1) [54]. These helicases are involved in various aspects of DNA repair, including **nucleotide excision repair** [55] and **methyl-directed mismatch repair** [56], as well as **replication restart** [57,58].

### UvrD-like SF1A monomers have a rotationally flexible regulatory (2B) domain

**Figure 2.**
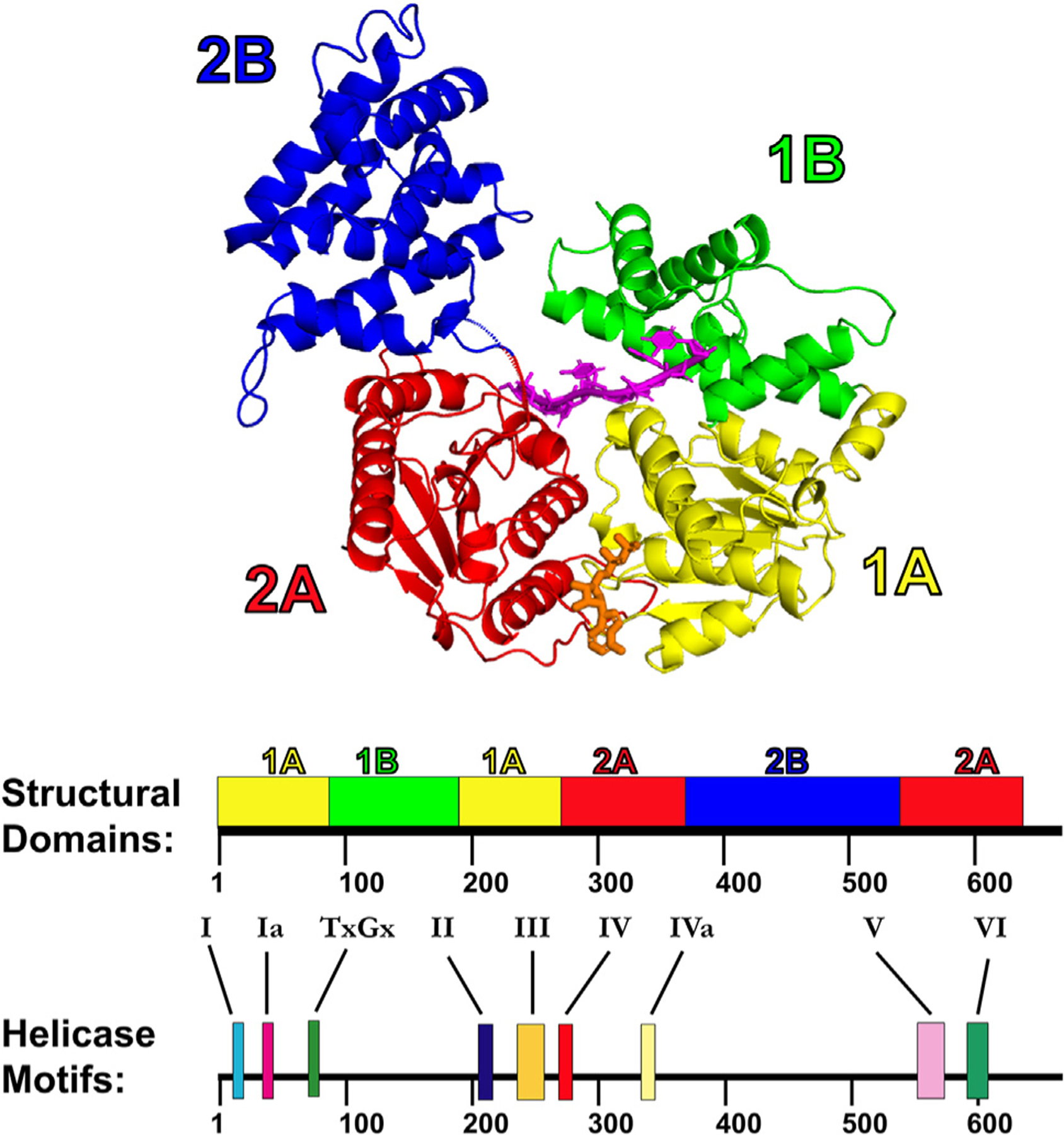
Crystal structure of the *E. coli* Rep monomer, a typical SF1A enzyme. *E. coli* Rep monomer crystal structure (PDB: 1UAA-chain B). Domains 1 and 2 consist of subdomains 1A (yellow), 1B (green), 2A (red), and 2B (blue). ssDNA (magenta) is bound above the 1A and 2A RecA (motor) subdomains, and ADP (orange) is bound between the 1A and 2A subdomains. The helicase motifs are contained within the 1A and 2A motor domains. SF1A: superfamily 1A; ssDNA: single-stranded DNA.

We focus here on the UvrD-like SF1A helicases, in particular, those found in bacteria. The general domain structure of the UvrD-like helicases is shown for *E. coli* Rep in Figure 2 [40]. These enzymes have two domains—each composed of two subdomains. The 1A (yellow) and 2A (red) subdomains (so-called RecA domains) are the core motor domains that are highly conserved in both SF1 and SF2 helicases [42]. The 1B (green) subdomain exits and re-enters the 1A subdomain, and the 2B (blue) subdomain exits and re-enters the 2A subdomain. All of the amino acids comprising the conserved signature ‘helicase motifs’ (I, Ia, TxGx, II, III, IV, IVa, V, and VI) are contained entirely within the 2 motor subdomains, 1A and 2A. The ATP-binding site occurs at the 1A/2A interface, and the ssDNA-binding site sits above the 1A and 2A subdomains. The 1B and 2B subdomains are auxiliary domains contained within Rep, UvrD, PcrA, Srs2, RecB, and UvrD1 but are less conserved among other SF1A helicases, such as human FBH1 [54]. As we discuss here, the 2B domain plays an important regulatory role, controlling DNA helicase activity. We also note that the Pif1-like helicases have 1B and 2B subdomains, although these differ from those of the UvrD-like helicases, whereas the Upf1-like helicases are missing these auxiliary subdomains [42]. These differences likely reflect differences in regulation, including possible interactions with other proteins.

**Figure 3.**
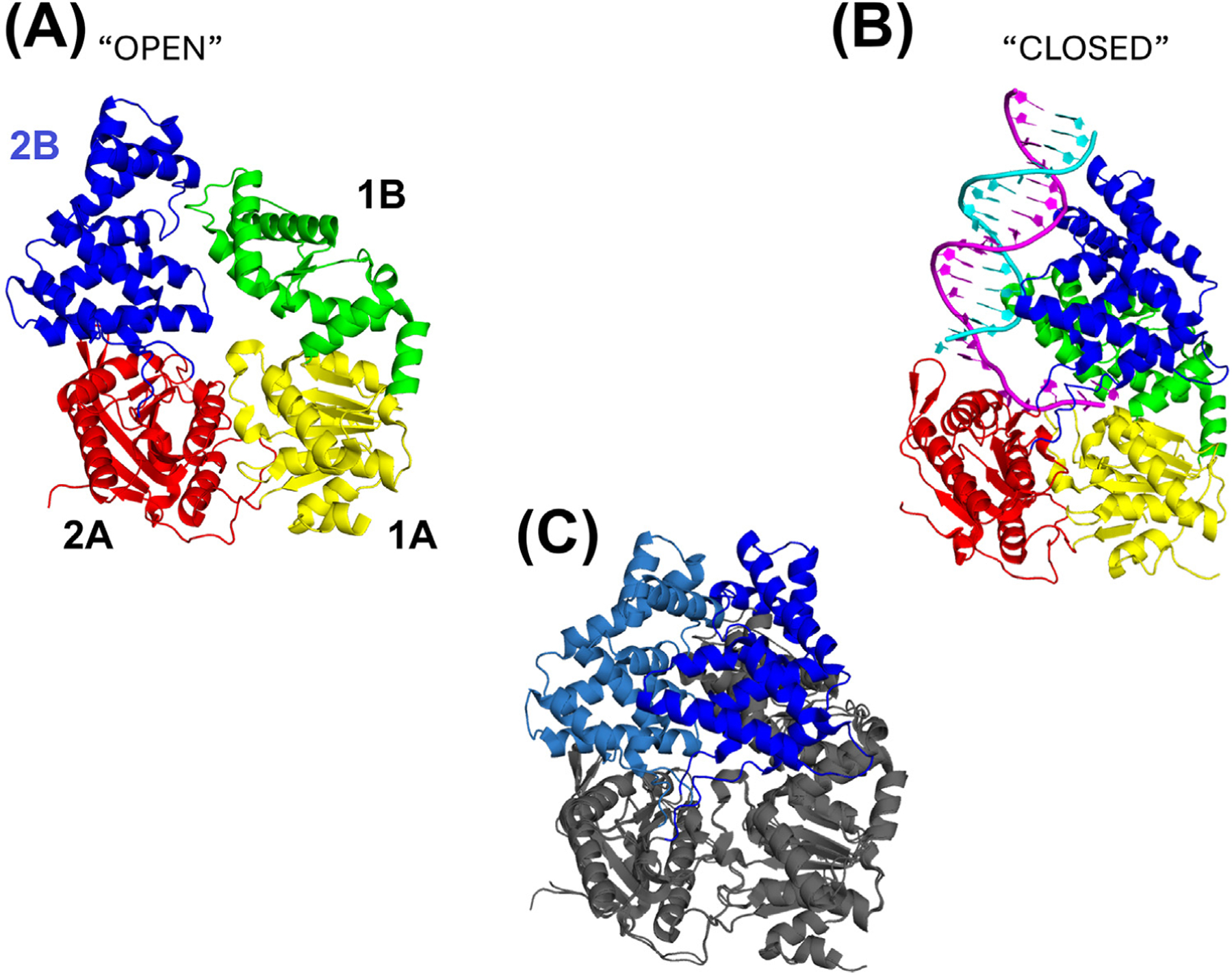
Two crystal structures of the *E. coli* UvrD monomer showing the large rotational conformational change of the regulatory 2B subdomain. (A) Crystal structure of the apo *E. coli* UvrD monomer (PDB: 3LFU) [29] with the 2B subdomain in the ‘open’ conformation. The GIG motif (magenta) and R421 (cyan) are indicated. (B) Crystal structure of *E. coli* UvrD monomer bound to an ss/ds DNA junction [3′-(dT)_7_–18 bp] (PDB:2IS2) [30] with the 2B subdomain in the ‘closed’ conformation and interacting directly with the duplex DNA. (C) Superposition of the closed (blue) and open (light blue) conformations of *E. coli* UvrD. The 2B subdomain rotates by ~160° in the transition from closed to open. GIG: glycine-isoleucine-glycine; ss/ds DNA: single-stranded/double-stranded DNA.

Crystal structures have been reported for the apo forms of PcrA [26] and UvrD [29], for Rep bound to ssDNA [28], and for PcrA [27] and UvrD [30] bound to short ss/duplex DNA junctions. Figure 3A shows the apo form of the UvrD monomer with the 2B subdomain in an open state, while Figure 3B shows a DNA-bound form of UvrD with the 2B subdomain in a closed state. Comparisons of these structures show that the 2B subdomains of all three proteins can undergo large rotations of 130–160° relative to the other three subdomains, as shown in Figure 3C for UvrD. This rotational flexibility has been demonstrated in solution in both ensemble [29] and single-molecule experiments [32,59,60]. Based on the large domain movements of the 2B subdomain of the Rep monomer, it was first hypothesized that dimerization might occur between the two 2B subdomains, enabling large relative movements between the two subunits that might contribute to the stepping of the dimeric enzyme along DNA [28]. In fact, the rotational conformation of the 2B subdomain plays a crucial role in enzyme activity and its regulation [15,28].

A major contributor to the controversy of whether the helicase activity of the UvrD-like family is due to monomers or dimers results from the fact that only monomers of SF1A helicases (PcrA [27] and UvrD [30]) bound to an ss/duplex DNA junction have been observed in crystal structures (Figure 3B). In both structures, the 2B subdomain is in a closed conformation and contacts the downstream duplex DNA. In fact, mechanistic models for how UvrD and PcrA monomers unwind DNA propose that the 2B subdomain–duplex DNA interaction plays a central role in the catalysis of DNA unwinding [27,30]. However, the ss/ds DNA junctions used in both of these structures possess only a seven-nucleotide ssDNA flanking region and cannot be unwound by either a UvrD monomer [8,16] or a PcrA monomer [20,25]. Hence, these monomer structures do not represent intermediates along the pathway to DNA unwinding.

### Functional studies demonstrate that UvrD-family helicases can be activated by dimerization

#### Dimerization of E. coli UvrD activates the helicase.

For any helicase that undergoes self-assembly, it is important to consider the protein and DNA substrate concentrations, as well as the solution conditions, when investigating enzyme activity [61]. It is also essential that the solution conditions and enzyme-to-DNA ratios that are used to characterize the assembly state of the enzyme–DNA complex are the same as those used to examine enzyme activity. Neglecting these issues has led to confusion concerning the functional form of the SF1A helicases.

**Figure 4.**
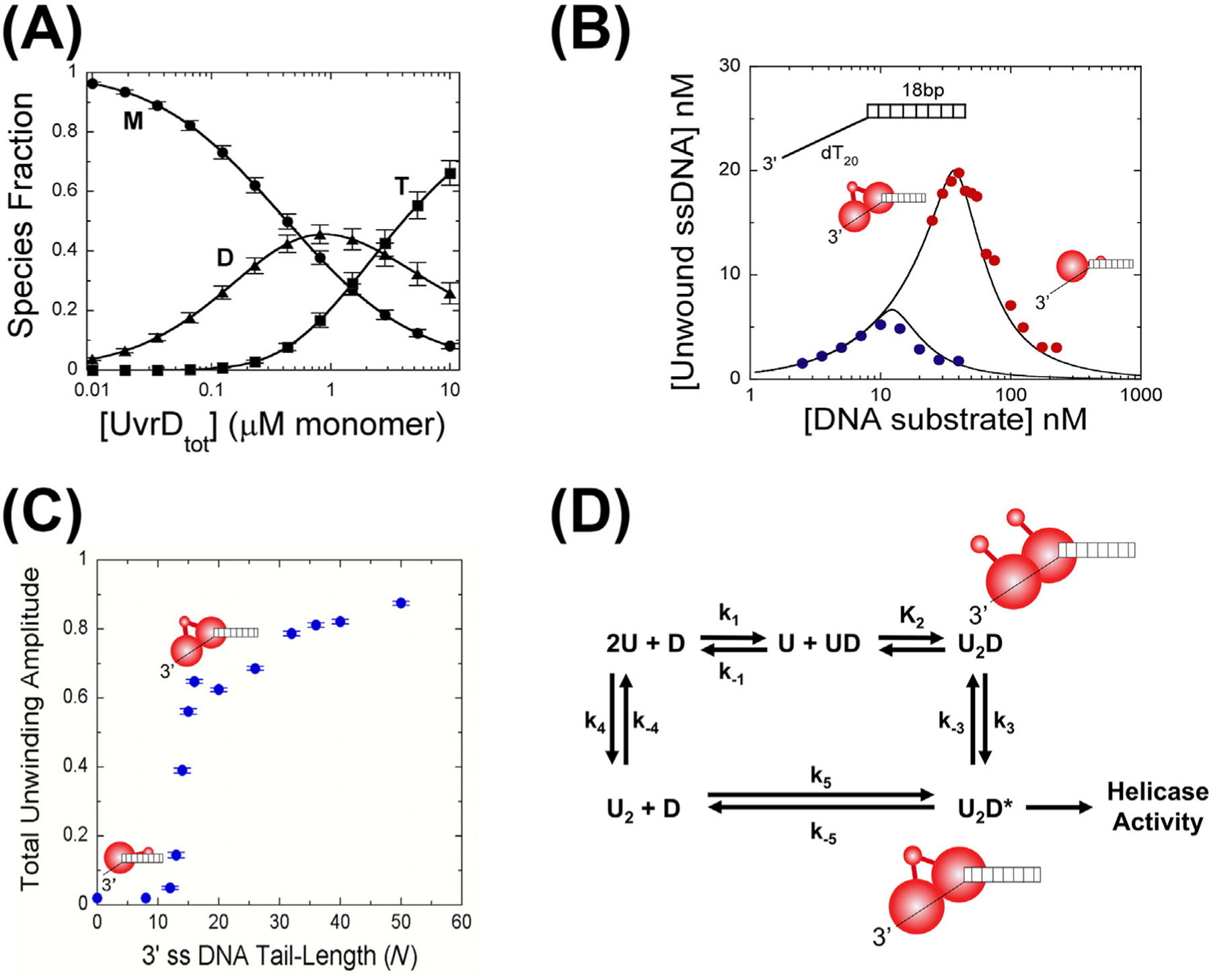
Self-association properties of *E. coli* UvrD. (A) *E. coli* UvrD exists in a monomer–(M)–dimer–(D)–tetramer–(T) equilibrium [62]. (B) The helicase activity of UvrD is dependent on the UvrD/DNA ratio. In excess protein, UvrD dimers bound to the DNA are favored and show helicase activity. In excess DNA, UvrD monomers bound to the DNA are favored and show no helicase activity [16]. 25 nM UvrD (blue); 75 nM UvrD (red). (C) UvrD helicase activity is dependent on the flanking 3′-ssDNA tail length (*N*). No helicase activity is observed for *N* ≤ 10 nucleotides as only UvrD monomers can bind to those DNA molecules. A sharp rise in helicase activity occurs for *N* ≥ 16 nucleotides because UvrD dimers can bind to those DNA molecules. (D) Helicase active UvrD dimers can assemble on a DNA substrate via two pathways: (top) two successive monomers can bind, followed by a conformational change (k_3_), or (bottom) a preformed dimer can bind directly to the DNA [63]. All experiments were performed at 25°C in 20 mM NaCl, 20% (v/v) glycerol, 10 mM Tris–HCl, pH 8.3. ssDNA: single-stranded DNA.

In the absence of DNA, *E. coli* UvrD exists as an equilibrium mixture of monomers, dimers, and tetramers [62] (Figure 4A). The equilibrium constants describing this system are sensitive to solution conditions, particularly salt concentration and type and glycerol concentration [62]. Hence, the helicase activity of *E. coli* UvrD is tightly linked to both protein and salt concentrations. Both UvrD monomers and dimers can bind to DNA; however, the distribution of monomers and dimers is affected by the protein/DNA ratio [16]. In particular, UvrD dimer–DNA complexes are favored at high UvrD protein-to-DNA ratios, whereas UvrD monomer–DNA complexes are favored when DNA is in excess over UvrD protein [16] (Figure 4B). This stems from the fact that UvrD monomers have a higher affinity for DNA than UvrD dimers, as well as the fact that UvrD-like dimers show negative cooperativity for DNA binding. Thus, high DNA-to-protein ratios lead to UvrD dimer dissociation, promoting monomer binding [16].

The length of the ssDNA tail of an ss/ds DNA junction also influences the binding of UvrD monomers versus UvrD dimers [16], as well as PcrA monomers versus dimers [25]. In particular, even at high UvrD-to-DNA ratios, DNA junctions with short (l < 10 nts) 3′-ssDNA tails favor UvrD monomer binding, whereas DNA junctions with longer 3′-ssDNA tails (l ≥ 26 nts) enable UvrD dimer binding (Figure 4C). As previous structural studies of both *E. coli* UvrD and *B. stearothermophilus* PcrA [27,30] utilized DNA junctions with 7 nt ssDNA tails, this ensured that only monomers would be bound to the DNA. In contrast, DNA junctions possessing longer 3′-ssDNA tails (l ≥ 20 nts) were used to examine helicase activity under conditions of excess protein over DNA, thus favoing UvrD and PcrA dimers [30,64]. Figure 4D shows the kinetic scheme for assembly of the active noncovalent *E. coli* UvrD dimer onto DNA via the monomer-to-dimer (top) pathway and the preformed dimer (bottom) pathway.

#### E. coli Rep helicase undergoes a DNA-induced dimerization.

*E. coli* Rep is monomeric in the absence of DNA, even at high concentrations (e.g., 8 μM) [65]. However, it undergoes DNA-induced dimerization when Rep is in excess over the DNA [65–69]. Just as for UvrD, under conditions of excess DNA, bound Rep monomers are again favored [66,67].

#### M. tuberculosis UvrD1 exhibits redox-dependent self-assembly.

Another variation on the theme of dimerization-based activation of helicase activity can be found in *Mtb* UvrD1 [21]. This enzyme contains a unique sequence motif in its 2B domain, completely conserved across mycobacterial species and found more broadly in a subset of Actinobacteria. More specifically, this motif contains a cysteine residue (C451) that uniquely drives covalent dimerization under oxidative conditions through the formation of a 2B–2B disulfide bond. Under oxidative conditions and low salt concentrations (i.e., <150 mM NaCl), higher-order oligomers are also observed. Analogous to the noncovalent dimers formed by other members of the UvrD subfamily, dimerization is needed for processive helicase activity. As such, under reductive conditions (e.g., the presence of 1 mM dithio-threitol (DTT)) or in the context of a C451A mutation, neither dimerization nor helicase activity is observed [21].

#### UvrD-family helicase activation can occur by pathways in addition to dimerization.

The helicase activity of the *E. coli* Rep monomer can also be activated via deletion of its 2B subdomain [19, 70,71]. This observation was the first indication that the 2B subdomain is regulatory (autoinhibitory) and does not play a catalytic role in DNA unwinding. This result also indicates that monomers possess all that is needed for helicase activity but are inhibited by the presence of the 2B subdomain [19]. This observation further supports the proposal that dimerization relieves autoinhibition by affecting the 2B subdomain rotational state and that the 2B subdomain might be directly involved in dimerization [19,28].

An intramolecular crosslink constraining the 2B subdomain in a relatively closed position (Rep-X) can also activate the Rep monomer helicase [72]. Interestingly, an internal crosslink that constrains the 2B subdomain to be in a relatively open conformation (Rep-Y) also stimulates DNA helicase activity over un-crosslinked Rep, but to a much lesser extent than Rep-X [72]. However, structures of these internally crosslinked monomers are not available, thus preventing a clear mechanistic interpretation.

Low processivity DNA unwinding by UvrD [73,74] and Rep [71] monomers has been observed in single-molecule experiments, but only when the DNA is under tensions of several piconewtons, which destabilize the DNA base pairs and lower the energy barrier for DNA unwinding. Interestingly, those studies also demonstrate a role for the rotation of the 2B domain of UvrD in regulating the DNA helicase activity of the monomer [73]. On the other hand, the application of force to the DNA appears to inhibit DNA unwinding by a UvrD dimer [75], leading to the proposal that an intermediate during DNA unwinding by the dimer involves some bending or compaction of the DNA [75].

## Resolution of the conundrum

The question of how dimerization activates the UvrD-family of SF1A helicases was recently answered when cryo-EM structures of a dimer of the *M. tuberculosis* (*Mtb*) UvrD1 helicase were obtained [31]. As mentioned earlier, *Mtb* UvrD1 helicase is also activated by dimerization, but in this case, the enzyme forms a covalent dimer via a disulfide bond between the same two cysteines (Cys451) in each of the 2B subdomains of each subunit [21]. Hence, helicase activation is redox dependent. Just as for the other SF1A enzymes, the *Mtb* UvrD1 monomer is capable of ATP-dependent 3′ to 5′ ssDNA translocation, but the monomer does not display helicase activity [21].

### Activation by dimerization results in a major rotation of the 2B subdomain

**Figure 5.**
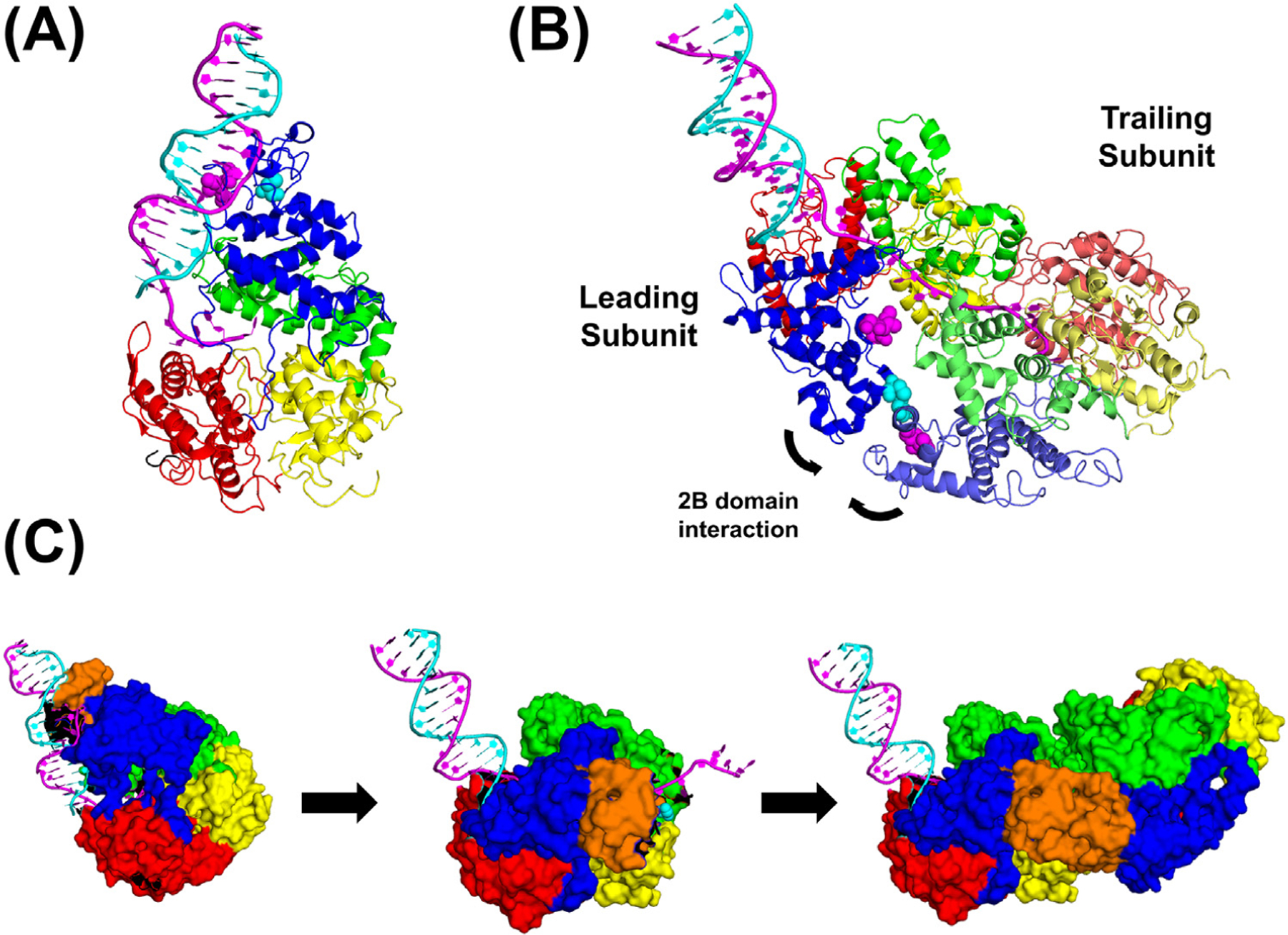
Transition of the *M. tuberculosis* inactive UvrD1 monomer–DNA complex to the active UvrD1 dimeric helicase involves a major rotation of the 2B subdomain. (A) Cryo-EM structure of the inactive *Mtb* UvrD1 monomer bound to a 3′-(dT)_10_–18 bp DNA junction (PDB: 9DGY). Note that the 2B subdomain (blue) interacts with the duplex DNA. Cys451 (cyan) is shown, as well as the GIG motif (magenta) that interacts with duplex DNA. (B) Cryo-EM structure of the active covalent *Mtb* UvrD1 dimer bound to a 3′-(dT)_20_–18 bp DNA junction (PDB: 9DES). Dimerization occurs between the two Cys451 residues (cyan) of the 2B subdomains of the leading and lagging subunits. Note that the 2B subdomain of the leading subunit no longer contacts the duplex DNA, and the GIG motif (magenta) has also moved away and no longer contacts the duplex DNA. (C) The transition of the 2B subdomain (blue) in going from the inactive UvrD1 monomer–DNA complex to the lead subunit of the active UvrD1 dimer to the active UvrD1 dimer bound to DNA. A region of the 2B domain that encompasses C451, which forms the disulfide crosslink between the two 2B domains in the UvrD1 dimer, is colored brown to highlight the change in its position relative to the duplex DNA after transitioning from the UvrD1 monomer–DNA structure. The same region of both subunits is highlighted in the UvrD1 dimer. ADP: adenosine diphosphate.

Cryo-EM structures of dimeric *Mtb* UvrD1–DNA junction complexes were solved along with a monomeric *Mtb* UvrD1–DNA junction complex [31] (Figure 5). The monomer UvrD1–DNA junction structure (Figure 5A), formed with a DNA junction possessing a (dT)_10_ ssDNA tail to only allow monomer binding, is nearly identical to the previously solved *E. coli* UvrD monomer–DNA junction structure [30] shown in Figure 3B. Specifically, the 2B subdomain is in its closed conformation and interacts directly with the duplex DNA in both structures, as it also does in the PcrA monomer–DNA junction structure [27].

In contrast, the dimeric *Mtb* UvrD1–DNA junction structure shows a major reorientation of the 2B subdomain of the subunit closest to the junction (leading subunit) (Figure 5B,C; Movie in the supplemental information online) [31]. The 2B subdomain of the trailing subunit (in the direction of unwinding) of the dimer is in the closed conformation previously observed in monomeric structures [30,31]. However, the leading subunit shows a unique 2B conformation not observed in previous structures. As a result, the 2B subdomain of the leading subunit no longer contacts the duplex DNA. In fact, the amino acid residues of the 2B subdomain that contact the duplex in the monomer–DNA structure (e.g., the GIG motif) are now located near the 2B–2B dimer interface in the *Mtb* UvrD1 dimer–DNA junction structure [31]. Hence, dimerization competes and is mutually exclusive with 2B–duplex DNA binding, strongly suggesting that the inability of UvrD monomers to initiate DNA unwinding is due to autoinhibitory 2B–duplex DNA interactions. Dimerization prohibits these interactions. This activation by dimerization that relieves the autoinhibitory interactions within the monomer is reminiscent of the activation of the EGF receptor dimer [76].

There are other major differences in the DNA interactions of the monomer–DNA junction complexes of UvrD [30], PcrA [27], and UvrD1 versus the UvrD1 dimer–DNA junction complex [31]. In particular, interpretation of the monomer–DNA crystal structures suggested that duplex DNA unwinding is facilitated by the presence of a pin or wedge region that serves to disrupt the DNA duplex during translocation by the monomer [27,30]. However, in the dimer structure, this same region is found nestled into a double-stranded DNA groove and is no longer in a position to serve in this capacity [31]. Consequently, the role of that region during unwinding [27,30] needs to be re-evaluated.

### *E. coli* UvrD dimerization uses the same 2B–2B interface as in *Mtb* UvrD1 dimers

The identification of the dimerization interface for the *Mtb* UvrD1 helicase raised the question of whether this interface is also used in other UvrD-family helicases. To examine this, a cysteine residue was engineered into the 2B subdomain of *E. coli* UvrD at the equivalent position of Cys451 of *Mtb* UvrD1. This *E. coli* UvrD mutant (R421C) is able to form a covalent, redox-dependent dimer that activates the UvrD helicase [31], indicating that the same 2B–2B interface is used to activate the *E. coli* UvrD helicase dimer. This result suggests that dimerization-dependent relief of autoinhibitory 2B–duplex DNA contacts formed by the monomer applies to *E. coli* UvrD and is thus likely a conserved mechanism across the UvrD subfamily.

### SF1A monomers can also be activated by accessory proteins that affect the 2B domain orientation

Although dimerization is needed to activate UvrD-family helicases *in vitro*, monomers can be activated through interactions with accessory proteins. This is consistent with the fact that a Rep 2B monomer [19] and an intracrosslinked Rep monomer (Rep-X) [72] both possess helicase activity, indicating that a monomer contains all that is needed for helicase activity.

MutL can activate a UvrD monomer helicase [32,33], PriC can activate a Rep monomer helicase [34], and RepD can activate a PcrA monomer helicase [35], all under single-round kinetic conditions. *Mtb* Ku can also activate an *Mtb* UvrD1 monomer, although this is only observed under multiple-round kinetics [51]. In the case of *E. coli* UvrD, this activation also involves movement of the 2B subdomain from a closed conformation to an intermediate conformation [32]. Furthermore, this activation appears to involve a direct interaction between the C-terminal region of MutL and the 2B subdomain of UvrD [36]. Hence, MutL activation also seems to involve relief of the autoinhibition of the 2B subdomain.

### Potential function for the autoinhibited SF1A monomer–DNA structures

Although the autoinhibited monomer–DNA junction structures of *E. coli* UvrD [30], *B. stearothermophilus* PcrA [27], and *Mtb* UvrD1 [31] do not appear to be relevant for DNA helicase activity, they may represent structures that function to prevent unwanted helicase activity by these monomers. An additional biological role of *E. coli* UvrD and *S. cerevisiae* Srs2 is as an antirecombinase. *E. coli* RecA–ssDNA filaments and yeast RAD51–ssDNA filaments are essential intermediates for DNA recombination events. Using their ssDNA translocase activities, UvrD monomers can disrupt RecA filaments within an ssDNA gap [77–81], and Srs2 can disrupt RAD51 filaments within an ssDNA gap [48,49,82,83]. Once the monomeric translocase reaches the 3′-ssDNA–duplex junction at the end of the gap, it may be important to prevent DNA unwinding beyond the gap. The direct interaction of the 2B subdomain with the DNA duplex at the junction would prevent unwanted helicase activity.

#### Evidence for UvrD dimer function in RNA polymerase backtracking during transcription.

Since helicase activation can occur via both monomer interactions with accessory factors and dimerization, the question remains as to how UvrD-family helicases are activated *in vivo*. It is likely that different activation pathways are utilized under different conditions and in different processes. Relevant to this issue, there is evidence that UvrD dimers are functionally important for *E. coli* RNA polymerase backtracking during transcription-coupled DNA repair [84,85].

## Concluding remarks

A detailed understanding of the mechanisms of DNA unwinding by any helicase sheds light on the complex machines that are critical components of all organisms. However, such knowledge can also facilitate their use as targets for therapeutic development (e.g., drugs, antibiotics, etc.) [86]. Targeting the dimer interface of UvrD-family helicases may offer one possibility.

The mechanisms of DNA unwinding proposed for UvrD-family helicases, based on the DNA junction complexes of monomeric *E. coli* UvrD [30] and *B. stearothermophilus* PcrA [27], need to be re-evaluated since these monomers do not possess helicase activity. Those models propose that the 2B subdomain–duplex DNA interactions observed in the crystal structures are essential for DNA unwinding, whereas this interaction is prohibited by dimerization in the dimeric UvrD1–DNA junction structure [31]. Although the 2B–duplex DNA interaction observed in the monomeric SF1A structures appears to be inhibitory, much remains to be learned about the functional interactions of the dimeric helicase during DNA unwinding.

Several mechanisms have been proposed that explicitly recognize the dimeric nature of the active UvrD-family helicase, with both subunits capable of interacting with both duplex and ssDNA [67, 68,75,87,88]. Most of the proposed models for how a dimeric helicase might unwind DNA invoke subunit-switching mechanisms in which the two subunits alternate their DNA-binding activities, regulated by the bound nucleotide state (e.g., ATP or ADP) [87]. These models are supported by studies showing that both subunits of the dimer are able to bind both ssDNA and duplex DNA individually as well as simultaneously [66,67,69,88–90]. There is also evidence for communication between the dimeric subunits [68,90–93]. Furthermore, since heterodimer formation between a wtUvrD subunit and an ATPase-deficient subunit is inactive, helicase activity requires more than rotation of the 2B subdomain alone [16,68].

**Figure 6.**
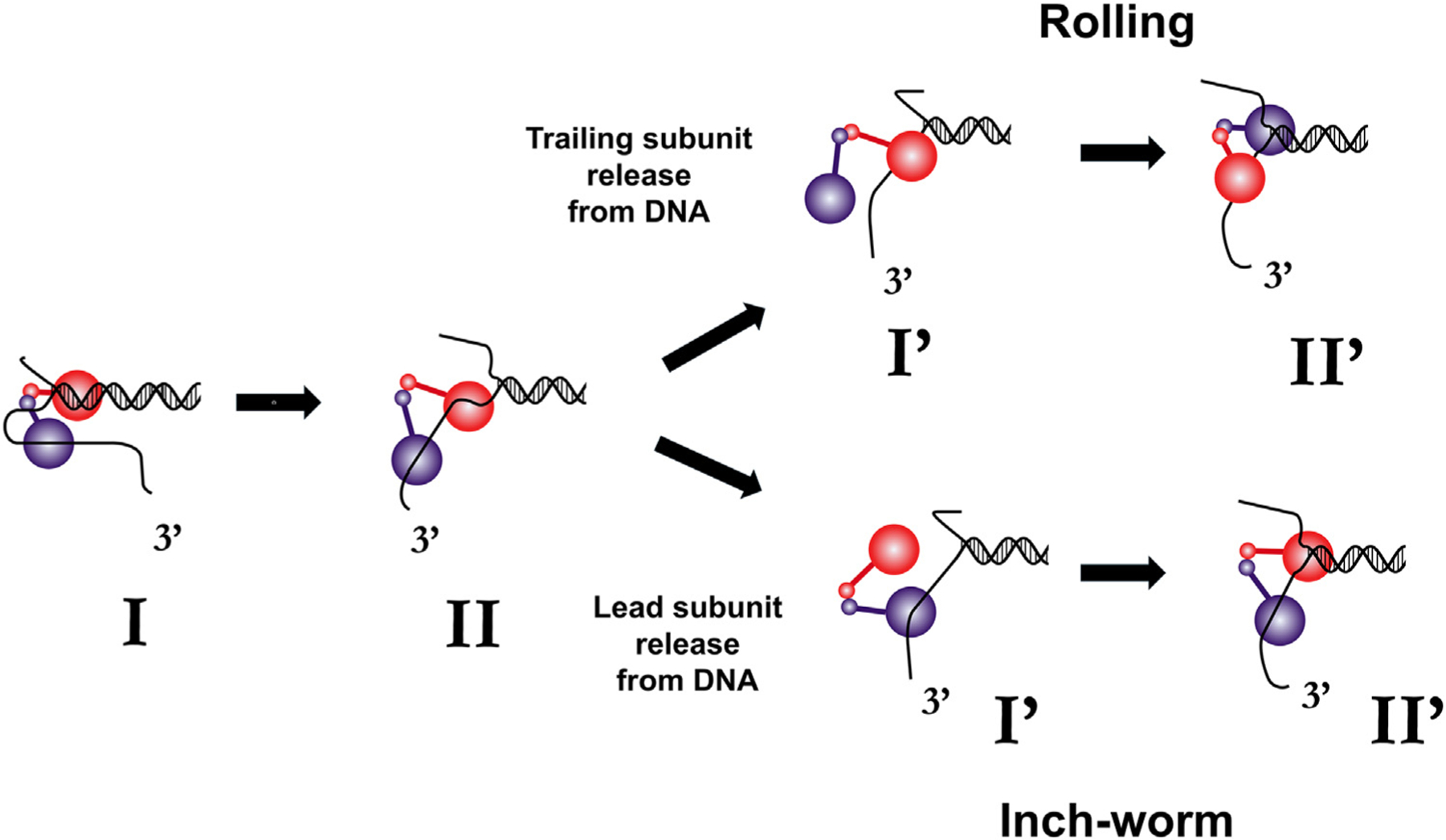
Potential mechanisms for DNA unwinding by a dimeric SF1A helicase. Dimeric rolling (hand-over-hand) model in which the leading and trailing dimer subunits switch positions during the DNA unwinding cycle [67]. Dimeric inchworm model in which the same subunit is retained as the leading subunit during the DNA unwinding cycle [68,87]. SF1A: superfamily 1A.

The first dimeric mechanism proposed was a **rolling model** (Figure 6), wherein each subunit alternates as the lead versus trailing subunit during the DNA unwinding cycle [67]. This model was based on the effects of nucleotides on the ssDNA and duplex DNA binding properties of the dimeric Rep helicase [67], in particular, the evidence that a Rep dimer is able to bind ssDNA and duplex DNA simultaneously [66,67,69,88–90]. The rolling model is equivalent to the hand-over-hand mechanisms that are used by kinesins for movement along microtubules [94].

Another plausible model for DNA unwinding by a dimeric helicase is the **dimeric inchworm** model [16,68,75] (Figure 6) in which the same subunit is maintained as the lead subunit through-out the unwinding cycle. Currently available experiments that have probed the DNA unwinding mechanism [67,88–92,95] do not distinguish between these two models. We know that both subunits need to be able to hydrolyze ATP [25,68] and that there exists a negative cooperativity for DNA binding to the two subunits that is influenced by nucleotide (i.e., ATP or ADP) binding [66,67], but many important questions remain regarding the mechanistic details of DNA unwinding (base pair melting) and translocation (see Outstanding questions).

Dimerization of UvrD-family SF1A helicases activates the helicase via relief of autoinhibition of a regulatory domain–duplex DNA interaction. This raises the question of whether other SF1/2 helicases require oligomerization for activation or regulation. In this regard, the SF2 DEAD box helicase HERA from *Thermus thermophilus* is known to function as a dimer with cooperative interactions between subunits [45]. The role of oligomerization in regulating the activity of other SF1/2 enzymes needs to be examined more generally and represents an important area for future studies.

## Supplementary Material

Lohman Movie

Supplementary information associated with this article can be found online at https://doi.org/10.1016/j.tibs.2026.02.005.

## Highlights

For the past few decades, the active form of superfamily 1A (SF1A) UvrD-family helicases has been controversial due to the absence of structures of the active dimeric form of these enzymes.

A key interaction in the monomeric structures is between a regulatory domain (2B) and duplex DNA that was proposed to facilitate DNA unwinding but is likely inhibitory.

However, recent cryo-EM structures show that *Mycobacterium tuberculosis* UvrD1 forms a covalent dimer, with dimerization occurring between the 2B domains of each subunit, resulting in major reorientations of the 2B domainsthatpreventthe2B–DNA interaction, thus relieving its inhibitory effect.

The same dimerization interface is used in *Escherichia coli* UvrD dimers, suggesting that this is a general mechanism to activate most SF1A helicases.

Due to these insights, textbook descriptions of helicase mechanisms based on the monomeric structures require re-evaluation.

## Outstanding questions

How does the dimeric helicase translocate along DNA? Does it use an inchworm or rolling (hand-over-hand) mechanism?

Does each subunit maintain continuous contact with the DNA during DNA unwinding and translocation, or is there an intermediate in which one subunit fully dissociates and the nreassociates with the DNA?

Do both subunits of the dimer interact only with single-stranded (ss) DNA, or does one subunit transiently interact with duplex DNA to facilitate base melting? Does the dimer interact with only one or both unwound ss DNA strands?

How are ATP binding and hydrolysis coupled to the mechanistic steps of base-pair melting and translocation?

How do the two subunits communicate during the DNA unwinding cycle?

Is activation by oligomerization used by other nonhexameric SF1 and SF2 helicases?

## Glossary

Accessory proteins: proteins that facilitate the function of helicase activity, either to initiate DNA unwinding or increase unwinding processivity.
Dimeric inchworm model: a model for DNA unwinding by a dimeric helicase in which the two subunits retain their roles as leading versus trailing subunits during the unwinding cycle.
Helicases: ATP-dependent enzymes that catalyze the separation (unwinding) of the two complementary strands of a duplex nucleic acid (DNA or RNA).
Methyl-directed mismatch repair: process by which DNA replication errors are repaired that are due to incorrect nucleotide incorporation during DNA replication.
Nucleotide excision repair: process of repairing DNA that has been damaged by environmental mutagens or UV light.
Processive enzymes: helicases that catalyze the separation of multiple base pairs of DNA in one binding event.
Replication restart: process by which a DNA polymerase can be restarted after it is stalled at a replication fork due to encountering a site of DNA damage.
Rolling model: a model for DNA unwinding by a dimeric helicase in which the two subunits alternate their roles as the leading versus trailing subunits during the unwinding cycle.
